# Medicine and Therapeutics

**Published:** 1897-11

**Authors:** William C. Krauss

**Affiliations:** Professor of nervous diseases, Medical Department, Niagara University


### MEDICINE AND THERAPEUTICS.

CONDUCTED BY WILLIAM C. KRAUSS, M. D.,
Professor of nervous diseases, Medical Department, Niagara University.

### EXTERNAL APPLICATION FOR PRURITUS.

THE aggravating itching of the skin in various parts of the body, of which many patients complain—a condition often unassociated with any discoverable pathologic state—can with almost absolute certainty be relieved with an ointment, as follows :

| | | | |
|---|---|---|---|
| ℞ | Mentholis................. | | 120 (gr. ii.) |
| | Adipis lanæ hydrosi ........ | 15 | (℥ss.) |
| | Adipis benzoinati.......... | 15 | (℥ss.) |

This gives almost instant relief. The menthol, in addition to its anesthetic action, also possesses germicidal action, and both of these probably are brought into play. Where an oily base is objectionable, alcohol may be used.— *Canada Lancet.*

### CONCLUSIONS DRAWN FROM FORTY-NINE CASES OF MALARIA IN CINCINNATI.

DRS. DUNHAM and Walden in studying these cases of malaria followed out a certain line of treatment, as follows :

1. Quinia was the only drug used, with the exception of iron and arsenic as tonics and hydrochloric acid as a placebo.
2. Generally two large doses were given, one four hours before and the other about the time of the chill, which were administered for several days and afterward small doses of two grains every four hours were given as a tonic.
3. In the treatment of the estivoautumnal form large doses of quinia were given regularly every four hours and hypodermic injections of quinia were used.
4. Comparatively small doses of quinine were sufficient.
5. Hypodermic injections of quinia proved effective after negative results had been obtained from quinia used in other ways.

---

### PARALYSIS OF THE ULNAR NERVE FROM BICYCLING.

DESTAT has communicated to the medical society of Lyons the following case of paralysis of the ulnar nerve, due to excessive bicycling : after a long ride the patient noticed a numbness of the little and ring fingers of the left hand ; also a paresis of the interossei, lumbricoides and adductor muscles of the thumb. These symptoms are due to a compression of the ulnar nerve against the pisiform bone, especially the superficial and deep palmar branches. The author refers to another case, reported by Simpson, of neuritis of the ulnar nerve, with atrophy of the muscles supplied by the nerve, following a long ride on a wheel by a young man who was only a beginner in bicycling.

---

### SENILE POLYNEURITIS.

STEIN calls attention in the *Münchener Medizinische Wochenschrift*, No. 12, 1897, to this form of polyneuritis, first described by Oppenheim, met with in the aged and characterised by the following phenomena : absence of all the etiological factors commonly met with in the various forms of polyneuritis ; presence, to a more or less marked degree, of arteriosclerosis ; chronicity of this form ; cranial nerves remaining unaffected ; absence of sensitive and irritative phenomena ; localisation in the lower extremities especially ; absence of vesical disturbances.

The author reports three cases of this affection, accompanied, however, with painful manifestations and vesical disturbances. In reaching a diagnosis the author warns against confounding it with tabes dorsalis, which may be suspected, inasmuch as the absence of patellar tendon reflexes are absent in both affections and Romberg's sign was present in one case. The ataxia is not present; neither are the eye symptoms. In one case, where the diagnosis was somewhat in doubt, the autopsy failed to show any lesion of the spinal canal. The autopsy of the three cases showed the presence of a cerebral apoplexy and a high degree of arteriosclerosis.

---

### TABES AND MOVABLE KIDNEY.

A. Habel (*Centralblatt für Innere Medizin*, February 20, 1897,) calls attention to the fact that in the literature only Eichhorst and Brieger have mentioned the occurrence of tabes dorsalis and movable kidney, and gives the results of his researches at the clinic at Zurich for 1895. In sixty-eight cases of tabes, movable kidney was found six times and always in women. Of all the other cases received at the clinic at the same period of time, only 1 per cent. of the women were affected with movable kidney.

The author believes that in tabes there exists a disposition to movable kidney, owing to the relaxed condition or deficiency of tone in the ligaments of the abdominal organs.

---

### NUTROSE IN DISEASES OF THE ALIMENTARY CANAL.

Dr. Appler, of Breslau, (*Therapeutische Monatshefte* for April, 1897,) describes the new preparation nutrose and gives his experience with the substitute for meat in cases of stomach and intestinal disorders.

Nutrose is a casein preparation prepared by the Hoechst Farbwerke, of Hoechst-on-Main. It is a white powder without any pronounced flavor or odor and is administered preferably in all kinds of soups (1 to $1\frac{1}{2}$ tablespoonfuls $= \frac{1}{3}$ to $\frac{1}{2}$ ounce in each plateful), milk, coffee, cocoa ($\frac{1}{3}$ ounce to each cupful), or, where less liquid and more solid food is indicated, together with pearl-barley, rice, oatmeal and the like ($\frac{1}{2}$ to $\frac{2}{3}$ ounce in each portion). In special cases nutrose mixes extremely well with various jellies or creams.

The indications for the employment of casein preparations, especially of nutrose, are manifold. Excluding for the present affections of the digestive organs, which will be considered separately, there present themselves in the front line consumptive diseases, such as tuberculosis and the like, cardiac and renal affections, cachexia from whatever cause, constitutional anemia or after hemorrhage and all affections in which it appears desirable to increase the albumen contents of the food imperceptibly. Likewise in the frequent repugnance exhibited to flesh and food in chlorosis, diabetes mellitus and the like, and in the convalescence of invalids, in order to convey to the system as much as possible easily assimilated food. Operations on the rectum, after which a less voluminous diet is desired, febrile diseases, *e. g.*, typhus abdominalis, in which principally only fluids can be taken, aftord further indications. Finally, the employment of the casein preparation is recommended in gout and uric acid diathesis, as we are thereby enabled to convey to the system the necessary quantity of albumen without the nuclein which makes meat a forbidden diet to the gouty patient—a postulate which was not fulfilled by the nitrogenous nutrient preparations hitherto known, made from meat.

The broadest field of employment for nutrose is, however, in the numerous diseases of the alimentary canal.

---

### THE TREATMENT OF ECZEMA WITH PICRIC ACID.

In an article on this subject in the *Nouveau Montpellier Médical* for September, M. A. Brousse remarks (*New York Medical Journal*) that the kerato-plastic property of picric acid, which has been successfully used in burns, seems to indicate that its employment is proper in the treatment of eczema, certain forms of which present great analogies to superficial burns. In 1889, he says, Cérasi employed this drug in seven cases of eczema with excellent results. Dr. McLennan, of Glasgow, was also very successful in the treatment of acute eczema and eczema of the face with this drug, which he used in a saturated solution. The author himself has obtained rapid recovery in several cases in which he has employed this treatment, the histories of which are given in detail. In cases of lichenoid eczema with a thick epidermis the acid was useless, but in acute oozing eczema accompanied by edema of the skin it was very useful. Under its influence in one case recovery was obtained in two weeks ; in another case, in ten days.

Among the advantages of this treatment are the immediate relief produced by the application of the picric acid solution and the disappearance of the pain, heat and itching ; the rapidity with which edematous tumefaction is effaced ; and the absolute painlessness of the dressing, even when it is applied to the bare surface of the derma. According to the opinion of the most competent observers, the extensive application of this drug does not give rise to any symptoms of poisoning. Not only is it useful in acute eczema, but it is also useful in the acute attacks of chronic eczema which are so frequent in arthritics, particularly if they are accompanied by oozing and ulceration of the skin ; it is equally useful in the seborrhoic eczema of infancy. The author states that the results obtained by him with this treatment absolutely confirm those indicated in the publication of Dr. McLennan, M. Gaucher and M. Leredde. M. Brousse therefore concludes that this treatment is indicated as follows : (1) In acute eczema ; (2) in the acute attacks of chronic eczema, particularly if there is a tendency to oozing and ulceration of the skin ; (3) in the seborrhoic eczema (impetiginous) of infancy. This treatment, he says, is contraindicated in chronic eczema and generally in all those of eczema which are accompanied by a thickening of the epidermis (lichenoid eczema). Nevertheless, it has the advantage, even in these cases, of allaying the itching.

---

(The following are all from the *Medical Review of Reviews.*)

### A THERAPEUTIC POTPOURRI.

Dr. Cattell gives the following brief résumé of the most recent advances in therapeutics (*Internat. Med. Mag.*, August, 1897) : Paraform pastilles may be purchased, which, by the aid of a small special apparatus, will quickly generate active formic aldehyde gas. An aqueous glycerin extract of the lungs of sheep is used by Brunet, both by the mouth and hypodermatically, in the treatment of pulmonary affections. Sodium copaibate, from ten to twenty-five grains, four, five or six times a day, is useful in the treatment of gonorrhea and is not so irritating to the intestinal tract and kidneys as corresponding doses of the oil of copaiba. Boric acid is of some value for the removal of the indelible yellow stains which sometimes remain upon the skin where burns have been treated with picric acid. When the skin has been burnt with carbolic acid, apply alcohol and wash in running water. The virulence of scarlet fever may be mitigated by large doses of carbolic acid frequently repeated, and it is said that any person taking scarlet fever from a patient who has been under this treatment will invariably have the disease in a milder form. For the paroxysms of angina pectoris Osler recommends nitrate of amyl in doses of from two to five minims, large doses of morphine hypodermatically and, when very severe, inhalations of chloroform. Thymus gland, eight grains three times a day, gradually increased to from fifty to 100 grains daily, is used in the treatment of exophthalmic goitre. Apioline has been used successfully in neurotic dysmenorrhea. An infusion of birch leaves acts as a diuretic. It is said that the Röntgen rays have been used with some success for the removal of hairy, pigmented moles. Stowell says that thyroid-gland extract is an excellent galactagogue. Gillette has found the injection of a teaspoonful of hydrogen dioxide an efficient remedy for epistaxis. Mariotti, after evacuating the contents of a tuberculous abscess, injects a 10 per cent. solution of oil of cloves suspended in sterilised oils. Chantemesse believes that the antitoxins may be injected into the rectum, after an enema, in the same doses and with as good results as when applied hypodermatically. Bienwald has arrested the hemorrhage of a small wound on the face of an hemophilic by the application of freshly-drawn blood from a healthy woman. Wright has had success with a solution of fibrin ferment and chloride of calcium as a styptic.

---

### COMPOUND TINCTURE FOR THE TREATMENT OF GOUT.

| | | | |
|---|---|---|---|
| ℞ | Tinct. stramonii | 4 | (ʒi.) |
| | Tinct. colchici | 6 | (ʒiss.) |
| | Tinct. guaiaci | 60 | (℥ii.) |

M. Three teaspoonfuls daily in milk.

—*Dr. V. Gayle.*

---

### TREATMENT OF SOFT CHANCRE BY SALICYLIC ACID OINTMENT.

A Hungarian practitioner, Dr. E. Szánto, has come to the conclusion that, of all the means employed against soft chancre, salicylic acid is the best. He uses it in the form of an ointment as follows:

| | | | |
|---|---|---|---|
| ℞ | Ac. Salicylic | 1 | (gr. xv.) |
| | Vaselin | 30 | (℥i.) |
| | Tinct. benzoin | 2 | (ʒss,) |

For external use.

The ulcer is first washed with a solution of corrosive sublimate and then covered with a piece of gauze, well charged with the salicylic ointment. With this treatment the chancre will heal much more rapidly than with any other, including iodoform ; and salicylic acid possesses the notable advantage of being quite odorless.

In the above formula the tincture of benzoin is added, not only to perfume the ointment, but also to increase its activity, as it has the property of holding the salicylic acid in a state of solution.—*Med. Week.*

---

### QUININE MIXTURE.

The following is advantageous in irritable stomach when quinine is to be given :

| | | |
|---|---|---|
| ℞ | Quinine sulph ............... | 12 (gr. ii.) |
| | Ac. citrici .................. | 36 (gr. vi.) |
| | Syr. simplici ............... | 035 (gr. ss.) |
| | Syr. aurantii flor ............ | 035 (gr. ss.) |

This is to be placed in a wineglass containing bicarbonate of sodium (from three to five grains) in saturated solution and drunk during effervescence.—*Jour. de Méd. de Paris.*

---

### SYRUP FOR THE TREATMENT OF EPILEPSY WITH VIOLENT EXCITEMENT.

| | | | |
|---|---|---|---|
| ℞ | Pot. bromidi ................. | 70 | (℥ii 2-5.) |
| | Pilocarpin nitratis ......... .... | | 035 (gr. ½.) |
| | Syr. amygdalæ amaræ....... | 400 | (℥xiii.) |
| | Aquæ.......................... | 600 | (℥xx.) |

A tablespoonful at a time.

Each tablespoonful of this syrup contains about a gramme of potassium bromide and half a milligramme of pilocarpin.

Under this treatment Dr. Voisin has found the maniacal excitement to cease and the diuresis to become more active. Gastric disturbances, due to the potassium bromide, are said to be less marked in such patients than usually, so that it is unnecessary to discontinue the treatment as frequently as is otherwise the case.—*Med. Week.*

---

### HYSTERIA DUE TO PELVIC DISEASE.

It is very common in women suffering from pelvic disease to find more or less pronounced symptoms of mental depression. They are brooding, despondent and frequently given to tears. For the relief of this condition, along with change of environment and directions for plenty of outdoor exercise, Dr. Talley found the following formula of service :

| | | |
|---|---|---|
| ℞ | Strychnin sulph ............ | 0026 (gr. $\frac{1}{42}$.) |
| | Quinin sulph............... | 063 (gr. iss.) |
| | Ext. hyoscyam............. | 063 (gr. iss.) |
| | Ferri redact............... | 06 (gr. i.) |

M. For one pill. Dose—One pill thrice daily.

—*Polyclinic.*

---

Cocaine poisoning is best treated by the recumbent position, amyl nitrite and aromatic spirits of ammonia, in water, slowly sipped.

---

FOR FEVER BLISTERS.

| | | | |
|---|---|---|---|
| ℞ | Camphoris................. | | 33 (gr. v.) |
| | Pulv. marantæ .......... ... | 2 | (gr. xxx.) |
| | Bismuth subnitratis ......... | 2 | (gr. xxx.) |
| | M. aquæ rosæ.............. | 16 | (ʒ iv.) |

---

RHEUMATISM.

For cases of acute or subacute rheumatism, in which there is considerable involvement of the muscles and also great pain, Dr. Eshner sometimes prescribes :

| | | | |
|---|---|---|---|
| ℞ | Phenazoni (antipyrin)....... | 8 | (ʒ ii.) |
| | Sodii salicylat.............. | 12 | (ʒ iii.) |
| | Ammonii bromid........... | 16 | (ʒ iv.) |
| | Aq. Cinnamomi ............ | 90 | (℥ iii.) |

M. Dose—A teaspoonful every three or four hours.

---

THE PREVENTION OF IODISM IN THE USE OF POTASSIUM IODIDE.

Spencer (*Journal de Médécine de Paris*) is credited with the following formula :

| | | |
|---|---|---|
| ℞ | Potass. iodidi....... ........ ...... | parts xxx. |
| | Ammon. ferrocitratis............... | parts iv. |
| | Tinct. nucis vomicæ............... | parts viij. |
| | Aq. distillatæ...................... | parts xxx. |
| | Tinct. cinchonæ............q. s. ad. | parts cxx. |

M. Sig. A teaspoonful in half a glass of water, to be taken after each meal. The tincture of nux vomica and the ammoniocitrate of iron are said to check the tendency to coryza and at the same time to act as tonics.

### ERYSIPELAS OF THE FACE.

THE *Presse Medicale* recommends the following formula :

| | | | |
|---|---|---|---|
| ℞ | Ac. carbolici.............. | | |
| | Tinct. iodini............... .. | | |
| | Alcoholis........................āā | 30 | (℥i.) |
| | Olei terebinthinæ............ | 60 | (℥ii.) |
| | Glycerini....................... | 90 | (℥iii.) |

M. : The lesions are to be painted with this liniment every two hours, and covered with aseptic tarlatan.

---

### AN APPLICATION FOR URTICARIA.

GAUCHER (cited in the *Gazette Hebdomadaire de Medicine et de Chirurgie* for July) recommends this :

| | | |
|---|---|---|
| ℞ | Mentholis........................... | 1 part. |
| | Chloroformi......................... | āā 3 parts. |
| | Etheris................................ | |
| | Spts. camphoris.................... | |

M. To be used as a spray or as a lotion. The part should then be dusted with powdered starch or zinc oxide.

---

### THE CREOSOTE TREATMENT WITH CHILDREN.

HOCK (*Wiener Med. Blat.; Centrabl. für Innere Med.*) uses creosote not only in tuberculosis, but also for persistent catarrhal phenomena after measles and whooping cough, according to the following prescription :

| | | | |
|---|---|---|---|
| ℞ | Creasoti........................ | 1 | (gr. xv.) |
| | Olei morrhuæ................ | 90 | (℥ iij.) |
| | Sacch ............................ | | 05 (gr. ¾.) |

M. From two teaspoonfuls to three tablespoonfuls to be taken daily.

---

DR. WEIL states in the *Practitioner* that every form of vomiting during gestation can be relieved by a 20 per cent. solution of menthol in olive oil. Dose, 10 drops on sugar whenever nausea appears."—*West. Med. Rev.*

---

### IODIDES AS TENICIDES.

ACCORDING to Dr. Newington, in the *Semaine Medicale*, the iodides appear to possess marked tenicidal properties. One of his patients unsuspectedly passed a large tapeworm after having taken the following mixture:

| | | | |
|---|---|---|---|
| ℞ | Pot. iodidi.... .............. | 2 | (gr. xxxv.) |
| | Iodini..................... | | 73 (gr. xiss.) |
| | Aquæ.......... . .......... | 30 | (fl℥j.) |

M. Sig. Ten drops three times daily.

Dr. Newington has subsequently had occasion to prescribe the above solution to several persons having tapeworms and in every instance the tenia was passed dead and no relapse ever occurred, it is reported.

---

### CHRONIC RHEUMATISM.

| | | | |
|---|---|---|---|
| ℞ | Potassii iodidi.... .......... | 12 | (ʒiij.) |
| | Vini colchici sem.... ....... | | |
| | Tinct. opii camph.........āā | 60 | (℥ij.) |
| | Tinct. stramonii.... ........ | 24 | (ʒvi.) |
| | Tinct. cimicifugæ.... ....... | 90 | (℥iij.) |

M. Sig. A teaspoonful thrice daily.—*St. Luke's Hospital, N. Y.*

—*Georg. Jour. of Med. and Surg.*

---

### ACUTE LOCALISED PROSTATITIS.

| | | | |
|---|---|---|---|
| ℞ | Iodoformi.................. | | |
| | Ext. hyoscyami...........āā | | 03 (gr. ss.) |
| | Olei theobromati .......... | 3 | (gr. xlv.) |

M. Sig. Use as a suppository.

—*Journal des Practiciens.*

---

### LUMBAGO.

| | | | |
|---|---|---|---|
| ℞ | Atropin........ .......... | | 25 (gr. iv.) |
| | Ac. oleic................. | 4 | (ʒi.) |
| | Olei ricini.... ............ | 4 | (ʒi.) |
| | Olei lavandulæ.......... .... | | 33 (mv.) |
| | Spts. rect............q. s. ad. | 30 | (℥i.) |

Sig. For local application.

—*Martindale and Westcott.*

---

### CANCER OF THE UTERUS.

| | | | |
|---|---|---|---|
| ℞ | Sodii chlorat......... ...... | 20 | (ʒv.) |
| | Syr. aurant. flor.... ........ | 30 | (℥i.) |
| | Aq. dest...... .............. | 90 | (℥iij.) |

M. Sig. From two to eight teaspoonfuls to be taken daily.

Locally :

| | | | |
|---|---|---|---|
| ℞ | Sodii chlorat................ | | |
| | Bismuth subnitrat āā........ | 5 | (ʒiiss.) |
| | Iodoform........ ........ .... | 2 | (ʒi.) |

M. Sig. Apply a small quantity on a tampon to the cervix.

—*E. Duvrae.*

